# Expression of HPV-16 E6 and E7 oncoproteins alters *Chlamydia trachomatis* developmental cycle and induces increased levels of immune regulatory molecules

**DOI:** 10.3389/fcimb.2023.1214017

**Published:** 2023-09-08

**Authors:** Carolina Olivera, Jessica P. Mosmann, Ailen N. Anna, Gloria N. Bettucci Ferrero, Daniela A. Paira, Fernando N. Ferreyra, María S. Martinez, Rubén D. Motrich, Cecilia G. Cuffini, Héctor Alex Saka, Virginia E. Rivero

**Affiliations:** ^1^ Centro de Investigaciones en Bioquímica Clínica e Inmunología (CIBICI), Consejo Nacional de Investigaciones Científicas y Técnicas (CONICET), Córdoba, Argentina; ^2^ Departamento de Bioquímica Clínica, Facultad de Ciencias Químicas, Universidad Nacional de Córdoba, Córdoba, Argentina; ^3^ Instituto de Virología “Dr. José M. Vanella”, Facultad de Ciencias Médicas, Universidad Nacional de Córdoba, Córdoba, Argentina; ^4^ Consejo Nacional de Investigaciones Científicas y Técnicas (CONICET), Córdoba, Argentina

**Keywords:** HPV, high-risk HPV genotypes, HPV E6 E7 oncoproteins, *Chlamydia trachomatis*, HPV-*Chlamydia* coinfection, immune inhibitory molecules

## Abstract

**Introduction:**

Infection with Human Papillomavirus (HPV) is a recognized risk factor for *Chlamydia trachomatis* (CT) infection and vice versa. Coinfection of HPV and CT in women is a very common and usually asymptomatic finding that has been linked to increased risk of cervical cancer. It has been demonstrated that CT facilitates the entry of multiple high risk HPV genotypes, leading to damage of the mucosal barrier and interfering with immune responses and viral clearance, which ultimately favours viral persistence and malignant transformation. Although the facilitating effects elicited by CT infection on viral persistence have been reported, little is known about the consequences of HPV infection on CT development.

**Methods:**

Herein, we took advantage of a genetically modified human cervical cell line co-expressing HPV-16 major oncogenic proteins E6 and E7, as an experimental model allowing to investigate the possible effects that HPV infection would have on CT development.

**Results and discussion:**

Our results show that CT infection of HPV-16 E6E7 expressing cells induced an upregulation of the expression of E6E7 oncoproteins and host cell inhibitory molecules PD-L1, HVEM and CD160. Additionally, smaller chlamydial inclusions and reduced infectious progeny generation was observed in E6E7 cells. Ultrastructural analysis showed that expression of E6 and E7 did not alter total bacterial counts within inclusions but resulted in increased numbers of reticulate bodies (RB) and decreased production of infectious elementary bodies (EB). Our results indicate that during CT and HPV coinfection, E6 and E7 oncoproteins impair RB to EB transition and infectious progeny generation. On the other hand, higher expression of immune inhibitory molecules and HPV-16 E6E7 are cooperatively enhanced in CT-infected cells, which would favour both oncogenesis and immunosuppression. Our findings pose important implications for clinical management of patients with HPV and CT coinfection, suggesting that screening for the mutual infection could represent an opportunity to intervene and prevent severe reproductive health outcomes, such as cervical cancer and infertility.

## 1 Introduction

Human Papillomavirus (HPV) and *Chlamydia trachomatis* (CT) are the most common causes of sexually transmitted infections worldwide (Smith and Angarone, 2015; Chen et al., 2020). Meta-analysis studies provided evidence on HPV infection risk in women infected with CT and CT infection risk in HPV-positive women, being each other reciprocal risk factors (Naldini et al., 2019). In addition, several reports have confirmed that the coinfection with HPV and CT is very common among asymptomatic women around the world (Bellaminutti et al., 2014; Ji et al., 2019; Ssedyabane et al., 2019). This relationship between urogenital CT and HPV infections seems to occur not only in females but also in males (Cai et al., 2014; Olivera et al., 2021). Some authors reported alterations in sperm quality in CT and HPV coinfected patients suggesting possible consequences on male fertility (Cai et al., 2014). Moreover, we recently reported a significant association between HPV and CT urogenital infections in male partners of infertile couples (Olivera et al., 2021).

HPV and CT infections share similar risk factors such as young age and a high number of sexual partners as well as common clinical features such as their asymptomatic nature favouring transmission (Nielsen et al., 2008; Skjeldestad et al., 2009). In addition, both pathogens are characterized by developing chronic persistent infections, possessing oncogenic properties and exerting different mechanisms of immune evasion that benefit themselves at expense of the host (Shew et al., 2013; Karim et al., 2018).

Immune evasion tactics utilized by CT include the entry into a persistent state in the presence of stressors, like exposure to cytokines or nutrient deprivation, which allows the bacterium to pause its developmental cycle and survive in an intracellular aberrant state for extended periods of time avoiding immune destruction and remaining able to resume normal propagation once the stressing condition is removed (Bayramova et al., 2018; Panzetta et al., 2019; Brockett and Liechti, 2021; Wang et al., 2022). In addition, CT can suppress the production of reactive oxygen species thus impairing immune cells phagocytic efficiency, manipulate intrinsic apoptotic pathways and induce the expression of host inhibitory molecules like PD-L1 (Wong et al., 2019).

HPV can also evade the immune system by different strategies through the expression of HPV oncoproteins E6 and E7, which take advantage of their high binding affinity to cellular immune regulatory proteins, blocking immune-related gene expression and immune signalling pathways and creating an overall immunosuppressive environment (Chiang et al., 2018; Zhou et al., 2019). Indeed, it has been reported that oncoproteins E6 and E7 can modulate Toll Like Receptor (TLR) signalling pathways, prevent the nuclear translocation of NF-κB and also interact with the major histocompatibility complex I promoter thus affecting antigen presentation and the subsequent induction of adaptive immune response (Li et al., 2009; Westrich et al., 2017; Barros et al., 2018).

Most individuals get infected with HPV during their lifetime and the immune system clears the virus within a couple of years post-infection (Chesson et al., 2014). However, 10-20% of infections persist for a longer time, increasing the risk of developing malignancies, indicating that other unidentified co-factors potentially drive the HPV-related disease outcome (McCloskey et al., 2017; Shanmugasundaram and You, 2017). Cofactors like immune status, hormones and coinfections with other pathogens have been postulated as causal links in cancer development (Jayshree, 2021). Certainly, high-risk human papillomavirus (HR HPV), including HPV-16/18, are strongly linked to the development of malignant transformation (Araldi et al., 2018). In addition, detection of CT was significantly associated with multiple HPV infections and HPV persistence (Silva et al., 2014). CT infections cause damage to the mucosal barrier, may interfere with immune response and viral clearance, resulting in inflammation, decreased effector T-cell population, dendritic cell activation and proinflammatory cytokine/chemokine production, thus favouring viral persistence. Additionally, CT infection causes chronic cervical inflammation and a decrease in lower genital tract antigen-presenting cells. In this way, CT infection results in an increased risk of coinfection with HPV, which may conduce to cervical cancer (Silva et al., 2014; Kumari and Bhor, 2022).

Although facilitating effects of CT infection on viral persistence have already been described, little is known about the potential consequences of HPV infection on CT development. Taking into account that HPV persistent infections *in-vivo* occur after the integration of viral genome into host epithelial cells producing stable E6 and E7 expression (Yeo-Teh et al., 2018), we mimicked a coinfection scenario by genetically manipulating an HPV negative cervical C33-A cell line to express HPV-16 major oncogenic proteins E6 and E7. Herein, we show that in cervical epithelial cells expressing HR HPV-16 E6E7 oncoproteins, CT developmental cycle is altered due to impaired RB to EB transition and infectious progeny generation associated with an upregulated expression of host immune regulatory molecules and E6/E7 oncoproteins.

## 2 Materials and methods

### 2.1 Cell culture and transfection assays

Cervical cancer-derived human epithelial cell lines C33-A (HPV-negative, ATCC #HTB-31), HeLa (HPV-18, ATCC #CCL-2) and Ca Ski (HPV-16, ATCC #CRM-CRL-1550) were maintained in Dulbecco’s modified Eagle’s medium (DMEM, Gibco) supplemented with 10% inactivated Foetal Bovine Serum (FBS) (Natocor), without antibiotics and incubated at 37°C in a 5% CO_2_ humidified atmosphere incubator. C33-A cells were transfected with the empty pLXSN vector (Clontech) or the one containing the HPV-16 E6/E7 ORFs (pLXSN16E6E7, Addgene plasmid #52394), kindly provided by Dr. Francisco Aguayo (University of Chile). Transfection assays were made using polyethyleneimine (PEI) as previously described (Masner et al., 2021). Briefly, one day before transfection cells were seeded onto six-well plates with 3 mL of basal MEM medium without antibiotics. The transfection mix (prepared by adding 2,5 μg of pLXSN or pLXSN16E6E7 recombinant plasmids, 125 μL 150 mM NaCl and 3 μL PEI) was incubated at RT for 15 minutes and then added to cells without antibiotics. Transfected cells were incubated for 24 h at 37°C in 5% CO_2_, washed with 1X PBS pH 7.4 and then incubated with DMEM supplemented with 5% FBS and 500 μg/mL of G418 for selection. Stably transfected cells (named C33-Empty or C33E6E7) were continuously propagated for 2-3 weeks under selection and frozen stocks were prepared. G418 selection was removed for at least 3 passages before experiments (10-14 days). To verify stable transfection, PCR assays targeting the HPV-16 *e6* and *e7* genes were performed using primers specified in **Table 1**.

**Table 1:** Used primers sequences.

Name	Forward primer (5’-3’)	Reverse primer (3’-5’)
HPV-16 *e6*	GAGAACTGCAATGTTTCAGGACC	TGTATAGTTGTTTGCAGCTCTGTGC
HPV-16 *e7*	GATGAAATAGATGGTCCAGC	GCTTTGTACGCACAACCGAAGC
TLR2	GGCCAGCAAATTACCTGTGTG	AGGCGGACATCCTGAACCT
TLR3	CCTGGTTTGTTAATTGGATTAACGA	TGAGGTGGAGTGTTGCAAAGG
TLR4	CCAGTGAGGATGATGCCAGAAT	GCCATGGCTGGGATCAGAGT
TLR5	TGCCTTGAAGCCTTCAGTTATG	CCAACCACCACCATGATGAG
TLR7	TTAACCTGGATGGAAACCAGCTA	TCAAGGCTGAGAAGCTGTAAGCTA
hBD1	ATGGCCTCAGGTGGTAACTTTC	TCGGGCAGGCAGAATAGAGA
GAPDH	GTGAACCATGAGAAGTATGACAAC	CATGAGTCCTTCCACGATACC

### 2.2 RNA purification, cDNA preparation and reverse transcriptase polymerase chain reaction for e6, e7, TLRs and hBD1

Cells in basal, stimulated or CT-infected conditions were trypsinized and used to extract RNA at the indicated times. Cellular RNA was isolated using the extraction commercial kit “Gen Elute Mammalian Total RNA Miniprep Kit” (Sigma) according to the manufacturer’s instructions. RNA integrity was determined by direct visualization of samples electrophoresed in 1% agarose gel in TAE buffer (40 mM Tris-Acetate, 1 mM EDTA). The RNA concentration and purity was determined using a Nanodrop 1000 spectrophotometer (Thermo Scientific). An aliquot of 2 μg RNA was treated with 1 μl of DNAse (1 IU/μl, Invitrogen) for 15 min at RT and denatured at 65°C. cDNA was synthesized in a 10 μL reaction volume containing DNAse-treated RNA (2 μg), 1U RNAse inhibitor (Promega), 2 μg of random primers (Biodynamics), 2 mM of each dNTP (Promega) and 10U of M-MLV Reverse Transcriptase (Promega). The reaction mixture was incubated for 1 h at 42°C followed by incubation at 65°C for 10 min. The cDNA samples were subjected to PCR amplification with specific primers for HPV-16 E6, HPV-16 E7, TLR2, TLR3, TLR4, TLR5, TLR7, hBD1 and GAPDH (**Table 1**). For cDNA amplification, 5 μL of the prepared cDNA were added to a tube containing 40 μM dNTPs (10 μM each); 10 μM of each primer and SYBR Green Master Mix (SIGMA). The PCR reactions were carried out using Step One Plus real time PCR equipment (Applied Biosystems) with the following cycling conditions: 10 min at 95°C, followed by 40 cycles of 95°C for 15 sec and 60°C for 1 min. Comparative C_t_ (ΔC_t_) was used as quantitation method. GAPDH amplification was used for data normalization.

### 2.3 Flow cytometry

Single cell suspensions containing 1x10^6^ cells were stained for surface markers. Dead cells were excluded using Live-Dead fixable reagent (Invitrogen). The following antibodies from eBioscience (San Diego, CA, USA) or Biolegend (San Diego, CA, USA) conjugated with appropriate fluorochromes were used: anti PD-1, PD-L1, HVEM, CD160, CD39, CD73, BTLA, and HLA-I. To assess E6 or E7 protein expression, indirect intracytoplasmic stain was performed after surface labeling. For this purpose, cells were fixed with Cytofix (BD) according to manufacturer’s instructions and subsequently permeabilized using Perm Wash permeabilization buffer (BD). Anti-E7 or anti-E6 Mouse IgG monoclonal antibodies were used. These primary antibodies were gently provided by Dr. Prat Gay from the Leloir Institute, Buenos Aires, Argentina (Ramirez et al., 2015). Then, goat anti-Mouse IgG Alexa Fluor^®^ 594 (Abcam) was used as secondary antibody. Finally, cells were acquired in FACS-CANTO II or LSR Fortessa flow cytometers (BD Biosciences, San Jose, CA, USA) and analysed using FlowJo™ v10.6.2 software. Proper compensation using Fluorescence Minus One (FMO) control was used.

For cell cycle analysis, cells were collected and fixed with 70% ethanol overnight at 4°C, resuspended with 50 μg/ml RNase A plus 50 μg/ml propidium iodide, and then acquired by FACS-CANTO II. Cell cycle data analysis was done using FlowJo™ v10.6.2 software.

### 2.4 Chlamydia trachomatis infection

For CT infection experiments, C33-Empty, C33E6E7 and Ca Ski cell monolayers were plated 24h before infection (in 6-well, 24-well or 96-well plates, as appropriate) in DMEM medium (Gibco) supplemented with 10% FBS (Natocor) without antibiotics, in a 5% CO_2_ humidified atmosphere at 37°C. Density gradient-purified EBs of CT serovar LGV-L2 strain 434/Bu ATCC VR-902B (L2 wt) or CT serovar E were obtained as previously described (Puerta Suarez et al., 2017). Infections were carried out by adding the appropriate amounts of previously titered EB suspensions of the specified CT strains to epithelial cell monolayers at the specified multiplicity of infection (MOI) in culture media, followed by centrifugation at 2,500 × *g*, 10°C, 30 min. Then, infected cells were transferred to a tissue-culture incubator and incubated in a 5% CO_2_ humidified atmosphere at 37°C for the indicated hours post infection (hpi).

### 2.5 Infectious progeny generation assay

To determine the generation of Inclusion Forming Units (IFUs), C33-Empty and C33E6E7 cells were seeded in 6-well plates and infected under the aforementioned conditions. At the time of infection, cell confluency was ∼80-90%. Forty hours later, cells were subjected to hypotonic lysis with water (800 μl/well) to release infectious particles and the appropriate volume of 5X sucrose–phosphate–glutamate (SPG) buffer (0.25 M sucrose, 10 mM sodium phosphate, 5 mM L-glutamic acid, pH 7.0) was added to obtain a final resuspension of the lysates in SPG 1X. Lysates were preserved at -80°C and later used to infect confluent monolayers of HeLa cells in order to assess the number of inclusion forming units (IFUs) per microliter as described (Nguyen and Valdivia, 2012; Panzetta et al., 2019). At 40 hpi, infected-cells were fixed for 15 min. with 4% paraformaldehyde and inclusions were visualized by indirect immunofluorescence-staining with rabbit anti-CT043 antibodies (Saka et al., 2011), followed by Alexa Fluor 468 goat anti-Rabbit IgG (Life Technologies). DAPI (Thermo fisher Scientific) was used to stain host cell and CT DNA. Imaging was carried out with Leica DMi8 microscope, and the analysis and counting was performed using ImageJ software (Schneider et al., 2012).

### 2.6 Inclusion size measurement

To determine CT inclusion size, C33-Empty and C33E6E7 cells were labelled with anti-CT043 antibody after 24 or 40 hpi as indicated above. The area of at least 100 inclusions per condition were measured and expressed as arbitrary units (a.u.) using the ImageJ software (Schneider et al., 2012).

### 2.7 Transmission electron microscopy

To evaluate the ultrastructural features of CT, monolayers of C33-Empty and C33E6E7 cells were obtained in 24-well plates and infected with CT (MOI:2). At 24 or 40 hpi monolayers were detached with a 0.5% Trypsin-EDTA solution for 30 sec. Subsequently, detached cells were centrifuged at 1500 rpm and the pellets obtained after centrifugation were fixed with Karnovsky fixative and stored at 4°C until processing. Samples were processed by the Electron Microscopy Center, School of Medical Sciences, National University of Cordoba. Post-fixation, the pellets were de-hydrated, embedded, cut, and examined as previously described (De Paul et al., 2009) using Zeiss Leo 906E TEM microscope. To enumerate the numbers of EBs and RBs, at least 30 inclusions were analysed per condition. Images were processed using the ImageJ software (Schneider et al., 2012) and Adobe Photoshop 2020 v21.0.3.91 (Adobe Systems Inc.).

### 2.8 In-vitro stimulation assay with TLR2 and TLR4 agonists

C33-Empty and C33E6E7 cells were incubated with the TLR2 ligand Zymosan (Sigma), and the TLR4 ligand LPS from *Salmonella* ser. Typhimurium (Sigma), at 100 μg/ml or 10 μg/ml, respectively, during 24h. After that, cells were trypsinized and RNA extracted to perform RT-PCR.

### 2.9 Statistics

All the experiments were done in triplicates and repeated at least three times. Statistical analysis was performed using the GraphPad Prism 9.1 software (∗*p* < 0.05; ∗∗*p* < 0.01; ∗∗∗*p* < 0.001; ns, not significant, *p* > 0.5). Tests performed are detailed in the corresponding figure legends.

## 3 Results

### 3.1 Generation of the C33-A cell line expressing HPV-16 E6 and E7 oncoproteins

**Figure 1:**
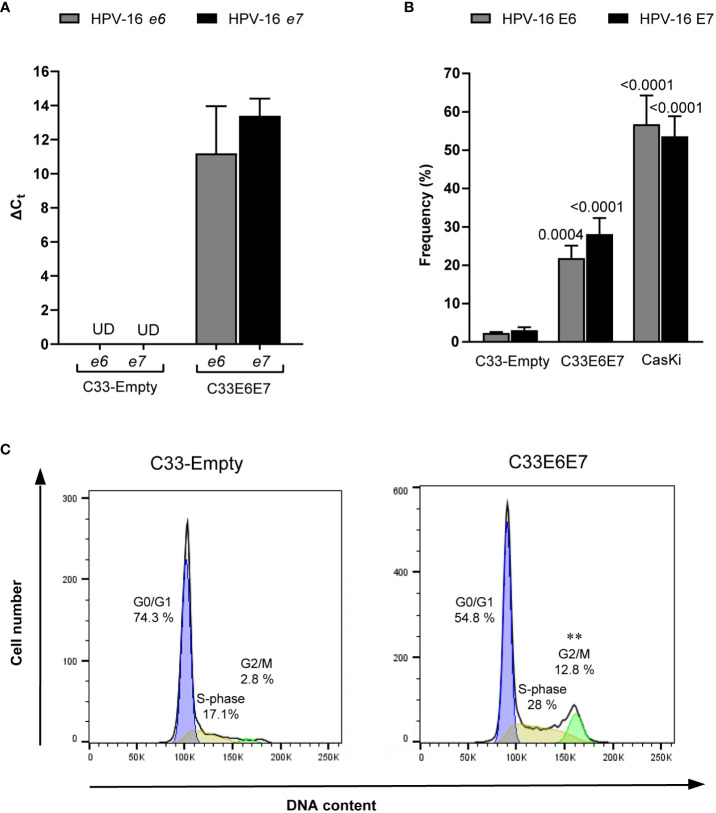
*Generation of a cell line stably transfected with HPV-16 e6 and e7 genes.* **(A)** Bar graphs showing relative mRNA expression of HPV-16 E6 and E7 analyzed by RT-PCR from RNA extracts of C33-Empty and C33E6E7 cells. Data are shown as mean ± SD of three independent experiments. Ct, cycle threshold; ΔCt, difference between Ct (gene of interest) and Ct (internal control); UD, undetectable. **(B)** Bar graphs showing E6 and E7 positive cells after intracytoplasmic staining with anti HPV-16 E6 or E7 mouse IgG monoclonal antibodies, as determined by flow cytometry analysis. Data are presented as the mean ± SD of three independent experiments. *p* values were obtained using unpaired *t*-test. Comparisons were performed between C33E6E7 or Ca Ski cells and C33-Empty (E6 or E7 as appropriate) used as control. A *p* < 0.05 was considered significant. **(C)** Representative histograms of flow cytometry analysis of DNA cell cycle after propidium iodide staining in C33-Empty and C33E6E7 cells. *p* values were obtained using unpaired *t*-test. Experiments were carried out in triplicate and repeated thrice. Comparisons were performed for each stage of the cell cycle for C33E6E7 vs. C33-Empty. **: *p*=0.001.

Transfection of C33-A cell line with a recombinant vector containing the HPV-16 E6 and E7 open reading frames allowed an efficient and stable expression of their corresponding transcripts (**Figure 1A**). E6 and E7 protein expression was also confirmed by intracellular staining with antibodies against E6 or E7 followed by flow cytometry analysis. Ca Ski cells, which contain multiple HPV-16 genomes, served as positive control. As expected, E6 and E7 protein expression was detected in both Ca Ski and C33E6E7 cells, but not in C33-Empty control cells (**Figure 1B**). Since HPV viral oncoproteins E6 and E7 modulate host cell cycle (Scarth et al., 2021), the progression of cells through the division cycle was assessed in C33-Empty and C33E6E7 cells by propidium iodide staining and flow cytometry. As expected, E6 and E7 protein expression in C33-A cells lead to a significant increase in cell frequencies in the G2/M phase (**Figure 1C**). These results indicate that HPV-16 E6 and E7 proteins are efficiently expressed and functional in our experimental model.

### 3.2 Innate immune receptors and *immunoregulatory* molecules expression in C33E6E7 cells

**Figure 2:**
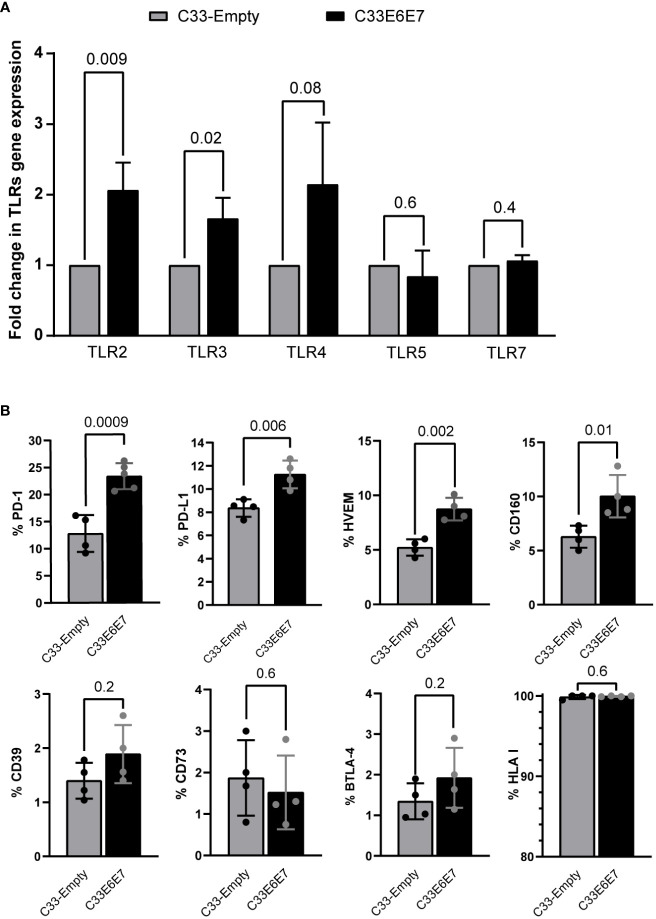
Expression of innate immune receptors and *immunoregulatory* molecules in cells expressing HPV-16 E6 and E7 oncoproteins. **(A)** Fold change expression of TLR2, TLR3, TLR4, TLR5 and TLR7 mRNA levels for C33E6E7 (black bars) with respect to C33-Empty cells (gray bars) analyzed by RT-PCR. Data are presented as the mean ± SD of three independent experiments. **(B)** Bar graphs showing percentage of cells positive for PD-1, PD-L1, HVEM, CD160, CD39, CD73, BTLA-4 and HLA I expression in C33-Empty or C33E6E7 cells, as determined by flow cytometry analysis. Experiments were done in triplicate and repeated thrice. Data are shown as the mean ± SD. Indicated *p* values were obtained using unpaired *t*-test as appropriate. A *p* < 0.05 was considered significant.

Previous reports indicate that E6 and E7 oncoproteins generate an overall immunosuppressive environment (Zhou et al., 2019). Thus, we investigated whether cells expressing these proteins displayed differential expression of TLRs and/or immunoregulatory molecules. RT-PCR was used to assess the expression of TLR2, TLR3, TLR4, TLR5, and TLR7 mRNAs in both, C33-Empty and C33E6E7 cells. Significantly higher expression levels of TLR2 and TLR3 were detected in C33E6E7 with respect to C33-Empty cells (**Figure 2A**). Moreover, a similar tendency was observed for TLR4 expression while no significant changes were detected for TLR5 and TLR7 (**Figure 2A**). Regarding molecules involved in immunoregulation, significant changes were observed between C33-Empty and C33E6E7 cells. Higher percentages of cells expressing immunoregulatory molecules PD-1, PD-L1, HVEM and CD160 were detected in C33E6E7 cells when compared to C33-Empty cells (**Figure 2B**). No differences were observed for the expression of CD39, CD73, BTLA-4 and HLA-I. These findings indicate that the expression of HR HPV-16 E6E7 oncoproteins elicits changes in immune molecules levels, upregulating the expression of activating receptors (TLRs) as well as regulatory molecules (PD-1, PD-L1, HVEM and CD160).

### 3.3 *Chlamydia trachomatis* development in a single infection or CT-HPV coinfection scenario

**Figure 3:**
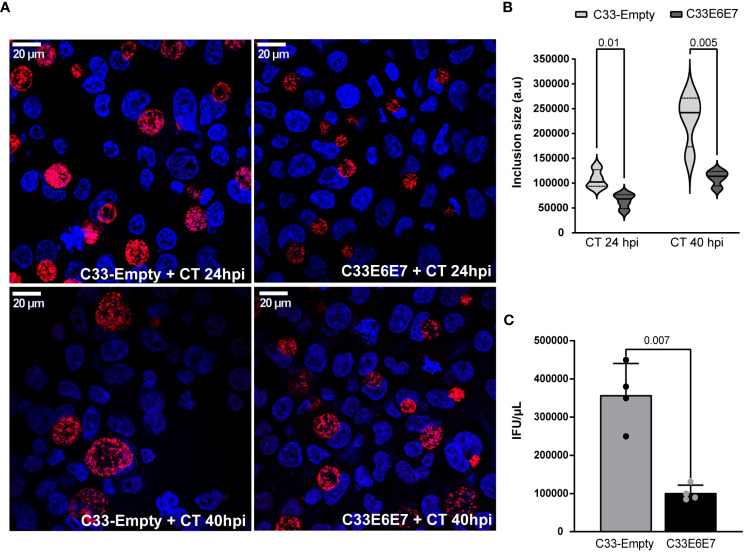
Chlamydia trachomatis development in cells expressing HPV-16 E6 and E7 oncoproteins. **(A)** Fluorescence microscopy micrographs of C33-Empty and C33E6E7 cells infected with CT (MOI: 0.5) at 24 or 40 hpi. Chlamydial inclusions were stained with rabbit anti-CT043 antibody followed by Alexa Fluor 468 goat anti-Rabbit IgG and DAPI. Confocal images are depicted. **(B)** Violin plots showing the determination of inclusion size expressed as arbitrary units (a. u.) in C33-Empty and C33E6E7 cells infected with CT at 24 or 40 hpi (black and dotted lines within violin plots indicate the median and interquartile ranges, respectively). At least 100 inclusions per condition were analyzed. **(C)** Quantification of CT infectious progeny generation in C33E6E7 (black bar) and C33-Empty cells (gray bar) at 40 hpi. The analysis is representative of at least three independent experiments. Data are presented as the mean ± SD. *p* values were obtained using unpaired *t*-test as appropriate. A *p* < 0.05 was considered significant.

To evaluate the interaction between CT and HPV-16 E6E7 proteins and the subsequent effect on CT development, E6 and E7 expressing (C33E6E7) or control (C33-Empty) epithelial cells were infected with CT LGV-L2 for 24 and 40 hours, as indicated. As shown in **Figure 3A**, the presence of E6 and E7 proteins affected the intracellular development of CT, since significantly smaller inclusions were observed in C33E6E7 cells with respect to C33-Empty cells at 24 as well as 40 hpi (**Figures 3A, B**). Experiments performed using CT serovar E yielded similar results (data not shown).

It is well known that CT has a biphasic developmental cycle involving non-replicative, infectious elementary bodies (EBs) and non-infectious replicative reticulate bodies (RBs) (Panzetta et al., 2018). Infection starts when EBs attached to the host cell membrane are internalized into a vacuole termed an “inclusion”. At early times post-infection, EBs transition to RBs, which replicate by binary fission as the inclusion expands. Around mid-cycle, RBs begin to asynchronically transition back into EBs, such that by the time of exit from the host cell, inclusions are filled with EBs able to initiate new infections in neighbouring cells (Elwell et al., 2016). In order to assess whether E6E7 expression affects CT developmental cycle, we performed infectious progeny generation assays by carrying out infections in our experimental model. EBs obtained from infected cells at 40 hpi were used to infect Hela cells monolayers and inclusions were enumerated. Interestingly, lysates obtained from infected C33E6E7 cells revealed a significant reduction of their reinfection ability, as reflected by a marked decrease in infectious progeny generation compared to C33-Empty cells (**Figure 3C**). These results indicate that the expression of HPV-16 E6E7 oncoproteins certainly interferes with CT development.

**Figure 4:**
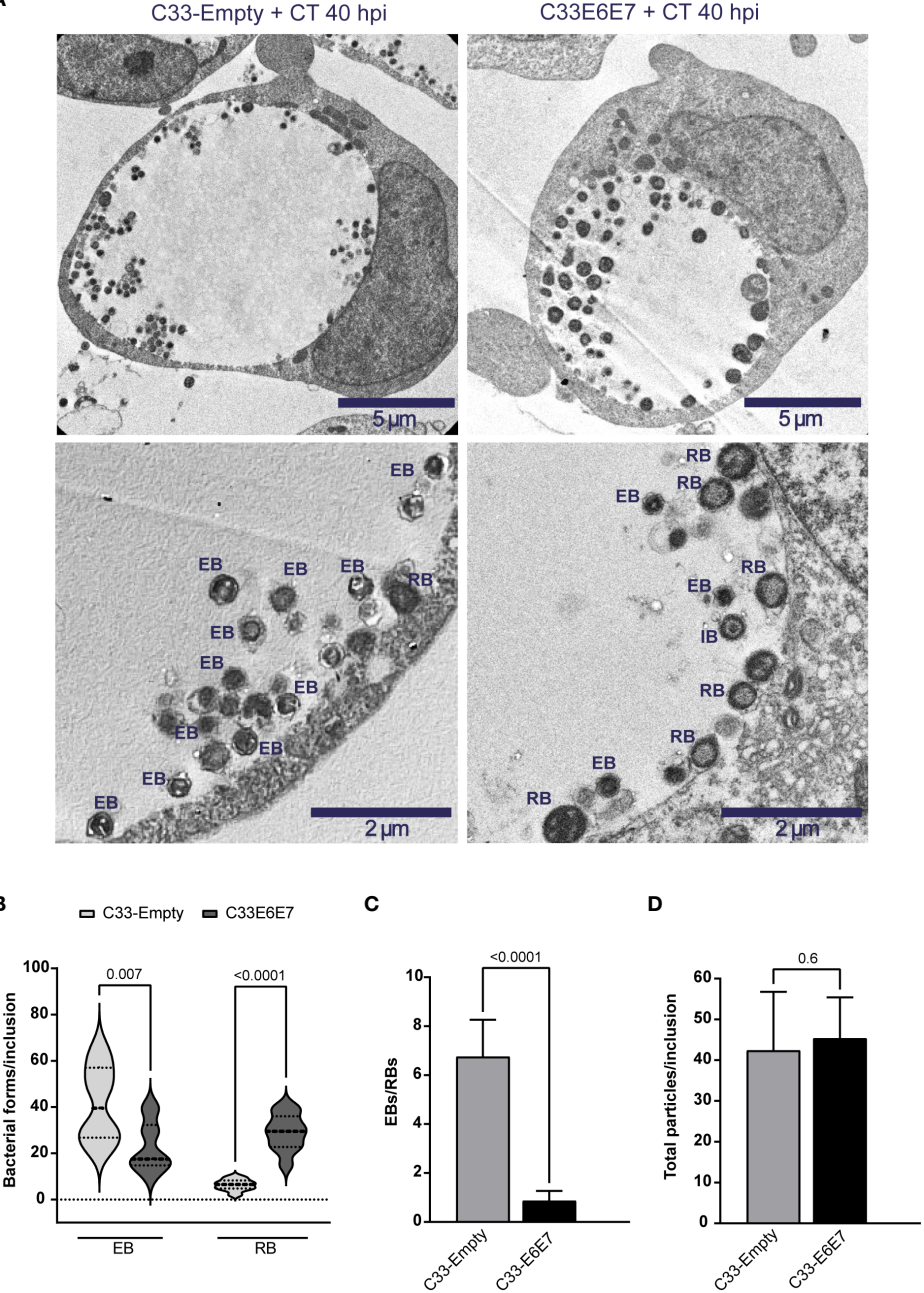
Ultrastructural analysis of Chlamydia trachomatis development in cells expressing or not HPV-16 E6 and E7 oncoproteins. **(A)** Representative transmission electron microscopy images of C33-Empty (left panel) and C33E6E7 (right panel) cells infected with CT at 40 hpi shown at 6000x (upper panel) or 15000x (lower panel) magnification. Chlamydial inclusions containing CT particles displaying typical ultrastructural features of reticulate bodies (RB), elementary bodies (EB) or intermediate bodies (IB) are indicated in lower pictures. **(B)** Violin plots showing the quantification of EBs and RBs per inclusion in CT-infected C33-Empty and C33E6E7 cells at 40 hpi (dotted lines indicate median and interquartile ranges). **(C)** EBs/RBs ratio in C33-Empty and C33E6E7 cells infected with CT at 40 hpi. **(D)** Number of total CT particles per inclusion in C33-Empty and C33E6E7 cells at 40 hpi. Bar graphs represent the mean ± SD of at least three independent experiments. At least 30 inclusions per condition were analyzed. *p* values were obtained using unpaired *t*-test as appropriate. A *p* < 0.05 was considered significant.

To evaluate the influence of E6 and E7 oncoproteins on different stages of CT development, we performed transmission electron microscopy of C33-Empty and C33E6E7 cells infected with CT LGV-L2. The ultrastructural analysis revealed that in both cell lines, inclusions contain a mixture of particles with typical morphological features of EBs, RBs and intermediate bodies (IBs), representing different CT developmental stages (**Figure 4A**). Quantification of the various developmental forms revealed that the proportion of EB and RB stages were significantly different in E6E7 positive (C33E6E7) and negative (C33-Empty) cells, while IBs were found in low numbers in both C33-Empty and C33E6E7 cells (1.2 ± 2.1 versus 1.4± 1.9, respectively, with no significant differences between them). Infected C33E6E7 cells showed lower quantities of small electron-dense EBs and higher numbers of larger and less condensed RBs when compared to C33-Empty cells at 40 hpi (**Figure 4B**). In agreement with this, a significantly lower EBs/RBs ratio was observed in inclusions of C33E6E7 compared to C33-Empty cells (**Figure 4C**). Interestingly, total numbers of bacterial particles per inclusion were not significantly different between cell lines strongly suggesting that CT is able to sustain replication in the presence of E6 and E7 oncoproteins (**Figure 4D**). Considering the non-infectious nature of RB as opposed to EB particles, this observation is in agreement with the decreased infectious progeny generation previously observed in C33E6E7 cells (**Figure 3C**). Overall, these results indicate that expression of HPV-16 E6 and E7 oncoproteins interferes with CT development by impairing normal RB to EB transitions rather than triggering a halt in bacterial replication or stressing conditions resulting in bacterial death.

### 3.4 *Chlamydia trachomatis* infection enhances E6 and E7 oncoprotein expression and impairs immune response in epithelial C33E6E7 cells

**Figure 5:**
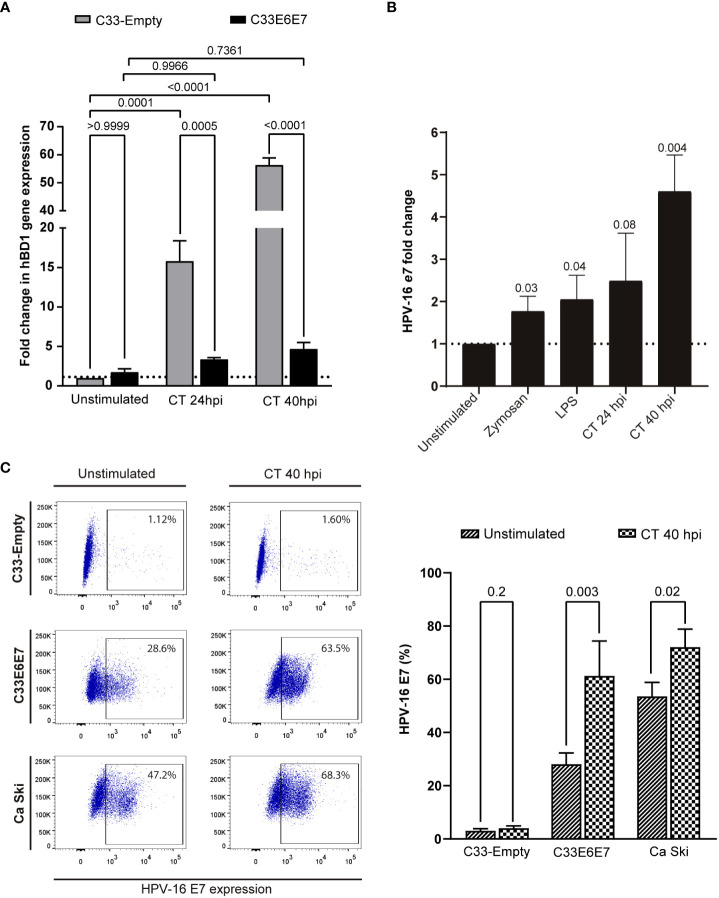
Chlamydia trachomatis infection enhances the expression of HPV-16 E6 and E7 oncoproteins. **(A)** Fold change expression of hBD1 mRNA levels for C33E6E7 infected or not with CT (24 or 40 hpi) with respect to CT-infected or unstimulated C33-Empty cells analyzed by RT-PCR. Data are presented as the mean ± SD of three independent experiments. Ordinary two-way ANOVA was performed. A *p* < 0.05 was considered significant. **(B)** Fold change expression of E7 mRNA levels for C33E6E7 cells stimulated with Zymosan, LPS or upon infection with CT at 24 or 40 hpi, with respect to unstimulated cells. Data are presented as the mean ± SD of three independent experiments. *p* values were obtained using unpaired *t*-test analysis as appropriate. A *p* < 0.05 was considered significant. **(C)** Representative dot plots (left) and bar graphs (right) showing E7 positive cells in C33-Empty, C33E6E7 and Ca Ski cells unstimulated and after 40 hpi with CT, as determined by flow cytometry analysis. Bar graph data are presented as the mean ± SD of two independent experiments. *p* values were obtained using unpaired *t*-test as appropriate.

**Figure 6:**
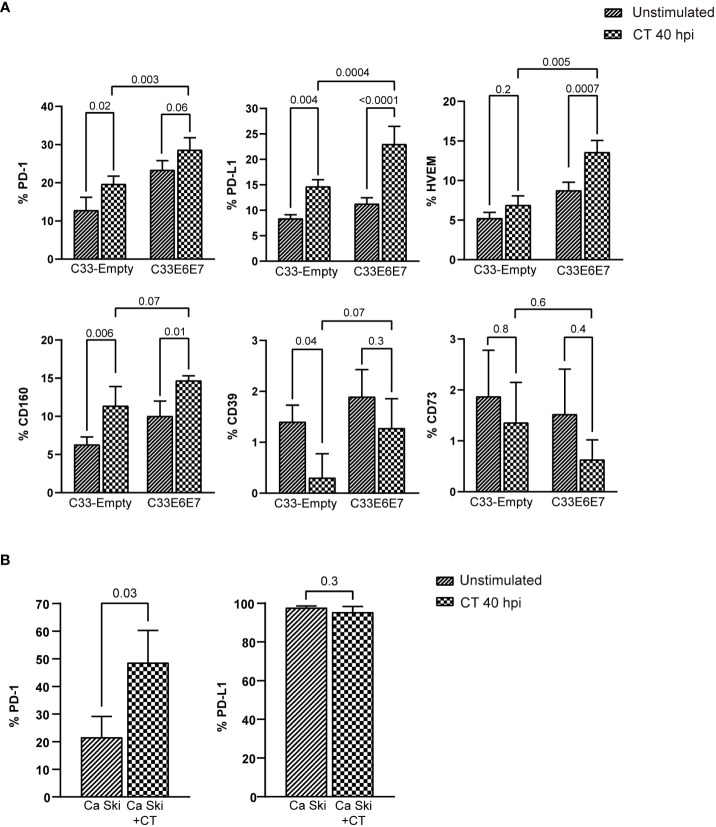
Chlamydia trachomatis infection enhances the expression of immune regulatory molecules. **(A)** Bar graphs showing percentage of cells positive for PD-1, PD-L1, HVEM, CD160, CD39 and CD73 expression in unstimulated or CT-infected C33-Empty or C33E6E7 cells at 40 hpi, as determined by flow cytometry analysis. Data are presented as the mean ± SD of three independent experiments. *p* values were obtained using Ordinary two-way ANOVA as appropriate. A *p* < 0.05 was considered significant. **(B)** Bar graphs showing percentage of cells positive for PD-1 and PD-L1 expression in unstimulated or CT-infected Ca Ski cells at 40 hpi, as determined by flow cytometry analysis. Data are presented as the mean ± SD of three independent experiments. *p* values were obtained using unpaired *t*-test as appropriate, a *p*<0.05 was considered significant.

Human beta-defensin 1 (hBD1) is an antimicrobial peptide that is produced by several epithelial cells after stimulation with micro-organisms (Erhart et al., 2011; Shelley et al., 2020). We wondered if E6E7 expression could affect C33-A cell´s ability to produce hBD1 upon CT infection. When hBD1 mRNA levels were compared in C33-Empty and C33E6E7 cells after 24 or 40 hpi, a significantly reduction was detected at both times analysed in C33E6E7 cells (**Figure 5A**). Noteworthy, in C33E6E7 cells, CT infection was able to induce increased levels of E7 mRNA mainly at 40 hpi (*p*=0.004). Although not significant, a similar trend was observed at 24 hpi (*p*= 0.08) (**Figure 5B**). Considering that TLR2 and TLR4 are important biosensors for CT recognition (Yang and Joyee, 2008), we then stimulated C33E6E7 cells with the TLR2 and TLR4 ligands Zymosan and LPS, respectively. As observed in **Figure 5B**, a significant increase in E7 expression was also detected after TLR2 and TLR4 stimulation. We then evaluated whether the effect seen at mRNA level was also observed at the protein level. As shown in representative dot plots and graph bars in **Figure 5C**, E7 expression changed in C33E6E7 and Ca Ski cells upon CT infection. Indeed, CT infection induces increased levels of E7 protein at 40 hpi in C33E6E7 cells (*p*=0.01), but not in C33-Empty cells. In addition, CT infection also induced increased E7 levels in Ca Ski cells, a cell line that has been reported to contain multiple copies per cell of integrated HPV-16 genomes (**Figure 5C**), indicating that changes in E7 expression upon CT infection are also observed when E6 and E7 are expressed under their natural promoter (Mincheva et al., 1987). Finally, we assessed whether E6 and E7 could affect the expression of immunoregulatory molecules (such as PD-1, PD-L1, HVEM, CD160, CD39 and CD73) by epithelial cells upon CT infection. After infection, a greater proportion of PD-1 positive cells was observed in C33-Empty cells with respect to unstimulated C33-Empty cells (*p*=0.02) (**Figure 6A**). Interestingly, C33E6E7 cells showed a high expression of PD-1 in unstimulated condition (**Figure 2B**), and a similar trend was observed upon CT infection (*p*=0.06). However, in CT-infected condition, significantly higher frequencies of PD-1+ C33E6E7 cells were observed compared to C33-Empty (p<0.003). Regarding PD-L1 and CD160, a similar effect was observed with significant increments in PD-L1+ cells and CD160+ cells in C33-Empty and C33E6E7 after CT infection. On the other hand, CT infection did not induce significant HVEM changes in C33-Empty with respect to unstimulated C33-Empty cells. Nevertheless, when C33E6E7 cells were infected with CT, significant increments in HVEM+ cells were observed (*p*<0.0007) (**Figure 6A**). In every case, the increment in these molecules’ expression (PD-L1, CD160, HVEM) was significantly higher in CT-infected C33E6E7 cells compared to CT-infected C33-Empty cells. Strikingly, CT infection induced a significant decrease in CD39 expression in CT-infected C33-Empty, while no changes were observed in C33E6E7 cells compared with their respective unstimulated conditions. No significant changes were observed in CD73+ cells upon CT infection in both cell lines (**Figure 6A**). Noteworthy, significant increments in PD1+ cells were also observed when Ca Ski cells were infected with CT (*p*=0.03), while a high frequency of PD-L1+ cells was observed in both unstimulated and CT infected Ca Ski cells showing not significant differences between unstimulated and CT infected conditions (**Figure 6B**). Based on these results, it appears that the presence of HPV E6 and E7 oncoproteins not only alters the normal cycle of CT leading to decreased inclusion size and impaired RB to EB transition, but also amplifies immune regulatory changes and increases the oncogenic potential already caused by HPV.

## 4 Discussion

Coinfections are experiencing an increasing interest in clinical practice nowadays. Although they have been increasingly detected, their implication in disease development and their consequences remain to be unveiled. Certainly, HPV and CT coinfection has shown a considerable prevalence being each other mutual risk factors of infection and cervical pathology (Samoff et al., 2005; Silva et al., 2014). Indeed, coinfection has been associated with increased risk for high-grade squamous intraepithelial lesions and higher CT DNA load (Ji et al., 2019). Remarkably, pre-existing chlamydial genital infection has been shown not to only increase the risk of acquiring HPV but also to favour viral persistent infection of HR and multiple HPV genotypes (Samoff et al., 2005; Silva et al., 2014). The evidence that CT may increase susceptibility to HPV infection or impair the efficient clearance of an existing HPV infection contributing to viral persistence, is particularly interesting (Zenilman, 2001; Silins et al., 2005). CT infection may function as entryway, allowing the access of HPV to basal epithelium layer (Paavonen, 2012). Moreover, CT can induce chronic inflammation (Kumari and Bhor, 2022) and squamous metaplasia, being metaplastic cells potential targets for HPV (Paavonen, 2012). In addition, CT may modulate the immune response favouring the persistence of HPV (Silva et al., 2014).

E6 and E7 oncoproteins from HR HPV act inhibiting tumour suppressors TP53, pRb and other molecular signals prolonging cell-cycle progression, delaying differentiation and inhibiting apoptosis in host cells (Moody and Laimins, 2010; Scarth et al., 2021). On the other hand, CT by itself has effects on cell cycle and proliferation. In fact, CT has been shown to alter the cell cycle, increasing cell proliferation and resisting apoptotic cell death in epithelial cells (Chumduri et al., 2013; Elwell et al., 2016; Challagundla et al., 2023). It has been reported that chlamydial infection also manipulates p53 isoforms, which also has a role on cellular proliferation (Challagundla et al., 2023). Thus, both pathogens act prolonging cell-cycle progression and inhibiting apoptosis. Herein, using an *in-vitro* model of coinfection, we demonstrate that during CT infection of C33E6E7 cervical epithelial cells and also Ca Ski cells, the expression of HPV-16 E6 and E7 oncoproteins increased even more suggesting that coinfection may have additive effects on cell cycle and cellular proliferation compared to each pathogen alone. In agreement, Challagunda et al. reported that CT infection of mouse epithelial cells expressing E6E7 increased E6E7 mRNA expression (Challagundla et al., 2023). Moreover, increased genital tract epithelial cell proliferation and cell cycle alterations were reported in both, *in-vitro* and *in-vivo* experimental models of CT infection and E6E7 overexpression, indicating that CT infection promotes cellular proliferation, survival and transformation (Kumari and Bhor, 2022). Interestingly, our results showed that stimulation of TLR2 and TLR4, important sensors for recognition of CT and many other pathogens, was also able to increase E6 and E7 expression (**Figure 5B**), indicating that coinfection of HPV and CT and possibly other coinfections may promote cell transformation (Wei et al., 2009).

On the other hand, it is well known that both HPV and CT manipulate and bias the immune system towards an immunosuppressive scenario (Wong et al., 2019; Zhou et al., 2019). Using an *in-vitro* model of coinfection, we found that CT infection induces a higher expression of the regulatory molecules PD-L1, HVEM and CD160 in cells expressing HR HPV-16 E6E7 oncoproteins, further supporting the additive effects of these pathogens. Augmented expression of PD-1 is considered indicative of T cell exhaustion as evidenced in many types of chronic viral infections (Golrokh Mofrad et al., 2020). PD-1 binding to its ligands (PD-L1 and PD-L2) induces cell anergy or apoptosis on activated T and B cells (Dyck and Mills, 2017). Liu et al. reported that HPV-16 E7 oncoprotein can boost the expression of PD-L1 hampering immunity against cancer. These authors found that overexpression of the E7 oncoprotein in human prostate epithelial cancer cells enhances PD-L1 expression and hinders peripheral blood mononuclear cell proliferation and function of cytotoxic T lymphocytes (Liu et al., 2017). It has been shown that normal cervical epithelium hardly expresses PD-L1; however, most cervical cancer cells display intense PD-L1 expression, being HPV positively correlated with increased levels of PD-L1 (Yang et al., 2013; Dyck and Mills, 2017; Liu et al., 2017; Chen et al., 2021). Herein, we demonstrated that CT infection also induces a higher expression of PD1 in Ca Ski cells and that almost 100% of them expressed PD-L1 in an unstimulated condition, supporting what has been reported by previously cited authors (Yang et al., 2013; Dyck and Mills, 2017; Liu et al., 2017; Chen et al., 2021). Moreover, PD-L1 has been shown to be also related to CT infection (Fankhauser and Starnbach, 2014). Indeed, in mice models of CT genital infection, a 10-fold higher expression of PD-L1 in the uterus was reported, a phenomenon that contributed to impaired bacterial clearance (Fankhauser and Starnbach, 2014). Thus, increased PD-1/PD-L1 expression has been reported in both HPV and CT infections. Our results support these data and show that coinfection with these two pathogens has additive effects, further favouring CT and HPV infection and persistence. Data from our work also demonstrated enhanced expression of HVEM and CD160 in epithelial cells expressing E6 and E7 oncoproteins after CT infection. HVEM is a human surface receptor of the TNF-receptor superfamily involved in signals communication between various cell types. It shows an extensive polygamous binding profile, serving as a pivotal switch in signal transduction by engaging four different ligands such as LIGHT, LTA, BTLA and CD160 (Liu et al., 2021). The molecular form and expression pattern of the ligand determines whether the interaction restricts or stimulates cellular activation. To our knowledge, there are no reports addressing these immune regulatory molecules in epithelial cells, neither in HPV infection or HPV-CT coinfection. Regarding CD39 and CD73, enzymes that participate in the production of immunosuppressive adenosine, some reports show expression of CD39 and CD73 in HPV positive cells and strong reduction after E6E7 silencing (Mora-García et al., 2017; Gutiérrez-Hoya et al., 2019; Mora-García et al., 2019). However, no reported data exists about CD39 and CD73 in the context of HPV-CT coinfection. In this way, our results reveal that C33E6E7 exhibited increased levels of immune modulatory molecules and that CT infection contributes to further upregulate their expression.

One of the most interesting results of the present work is the observation that expression of HR HPV-16 E6E7 oncoproteins in C33E6E7 cells altered CT development. Indeed, the presence of E6 and E7 proteins resulted in smaller CT inclusions and a significant reduction in infectious progeny generation. Additionally, ultrastructural analysis of CT-infected C33E6E7 cells revealed a decreased EB/RB ratio at the end of the CT developmental cycle, while the total number of bacterial particles was not significantly reduced compared to C33-Empty cells. These findings indicate that while HPV E6E7 expression does not seem to abolish CT replication, it clearly impairs RB to EB transition. All these observations are in agreement with a recent report by Koster et al., indicating that HPV-16 E6E7 expression interferes with the normal development of CT in a human-derived ectocervical organoid model of HPV-CT coinfection (Koster et al., 2022). In fact, these investigators found that HPV-16 E6E7 expression resulted in reduced inclusion size, impaired infectious progeny generation, lower counts of EBs per inclusion and increased RBs per inclusion. Based on their findings, Koster et al. proposed that HPV may induce CT persistence. Interestingly, it has been demonstrated that CT persistence induced by tryptophan starvation was different in HPV-positive or HPV-negative cell lines. In this sense, Sherchand et al. demonstrated that C33-A cells generate sufficient intracellular tryptophan via proteasomal activity to permit CT replication, while C33E6E7 cells behave like HeLa cells during tryptophan starvation allowing CT persistence (Sherchand et al., 2016). Remarkably, tryptophan starvation protects CT from clearance by doxycycline in HPV-positive cells but not in HPV-negative cells (Sherchand et al., 2016). These results support that the presence of E6E7 proteins or HPV coinfection may favour persistence induction upon essential nutrients deprivation. Importantly, CT persistence has already been demonstrated in another viral coinfection scenario. Certainly, coinfection of herpes simplex virus (HSV) type 2 with CT led to chlamydial persistence (Deka et al., 2007). Using *in-vitro* coinfection models, it was found that the interaction of an HSV glycoprotein was sufficient to trigger both suppressed production of infectious chlamydial progeny and stimulated formation of swollen, aberrantly shaped RBs (Deka et al., 2007; Vanover et al., 2010).

**Figure 7:**
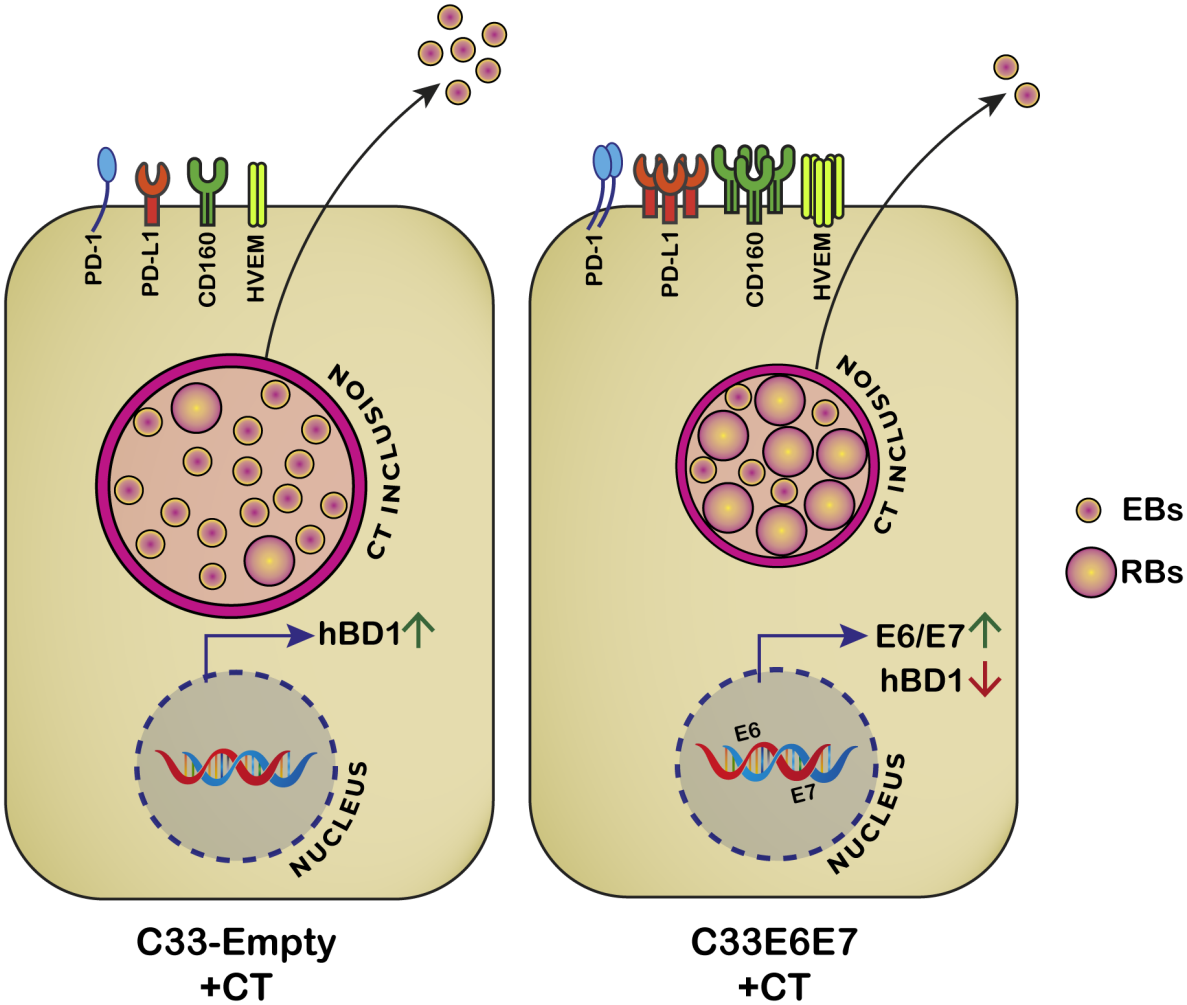
Proposed model for HPV-16 E6 and E7 oncoproteins effects on *C. trachomatis* development and immunoregulatory molecules. When CT infects C33E6E7 cells, smaller inclusions are produced and RB to EB transition is impaired. Consequently, fewer infectious particles are released at the end of the developmental cycle. Simultaneously, increased expression of E6 and E7 oncoproteins and the immunoregulatory molecules PD1, PD-L1, HVEM and CD160 as well as decreased levels of hBD1are observed.

A summary scheme illustrating a proposed model for HPV-16 E6 and E7-induced changes in CT developmental cycle and immune regulatory molecules is shown in **Figure 7**. Although HPV E6 and E7 proteins have been the subjects of intense research over the past few decades, there are still some surprising gaps in our understanding of how they work, especially in a coinfection scenario. In this context, the precise molecular mechanisms underlying the effects of HPV-16 E6E7 expression on CT development and persistence are unclear and need further investigation. Understanding the mechanistic basis of these effects could be important in developing strategies for prevention and treatment of cervical cancer and other related conditions. Our results add to the current knowledge in the field supporting the idea that during CT and HPV coinfection both tumorigenesis and immunosuppression are potentiated since during the coinfection inhibitory molecules are highly expressed and HPV-16 E6E7 oncogenes are co-operatively enhanced. It is tempting to speculate that these oncogenes may induce CT entry into a persistent-like stage in which the bacteria can evade the immune system. Thus, any alteration in the host that leads to transient immunosuppression and reactivation of a latent HPV virus infection will also influence the course and consequences of a CT infection. These findings bear important implications for future screening and management of patients with HPV and CT coinfection. In women diagnosed with HPV or CT infections, the screening for the mutual infection could represent a preventive intervention for severe reproductive health outcomes such as cervical cancer and infertility.

## Data availability statement

The raw data supporting the conclusions of this article will be made available by the authors, without undue reservation.

## Author contributions

VR and HS obtained funding, conceived and designed the experiments. CO substantially contributed to the conception and design of the work, acquired, analyzed, and interpreted the data. CO, JM, AA, GB, DP, FF and MM performed experiments. CO, VR, CC, JM, RM, HS drafted and critically revised the manuscript. All authors contributed to the article and approved the submitted version.
